# Aftermath of Chlamydia Trachomatis – The Tip of an Iceberg in Female Reproductive Health

**DOI:** 10.34763/jmotherandchild.20232701.d-23-00033

**Published:** 2023-10-16

**Authors:** Aparnna Vaikundam Subramanian, Sruthi Nagarajan, Poongodi Santhana Kumarasamy

**Affiliations:** Government Primary Health Centre, Pavoorchatram, Tamil Nadu, India; Department of Obstetrics and Gynecology, Tirunelveli Medical College, Tirunelveli, Tamil Nadu, India; Department of Microbiology, Tirunelveli Medical College, Tirunelveli, Tamil Nadu, India

**Keywords:** Infertility, Chlamydia trachomatis, prevalence, PCR

## Abstract

**Objective:**

The magnitude of infertile couples worldwide was found to be 60–80 million. Genital infection due to Chlamydia trachomatis (C. trachomatis) is one of the most prevalent sexually transmitted infections (STIs) which may present as PID, leading to ectopic pregnancy, infertility or other adverse health outcomes. This study was done to assess the prevalence of C. trachomatis infections among female patients with infertility using real time PCR (RT-PCR) and to compare the findings of molecular testing with hysterosalpingography (HSG) and ultrasonography (USG).

**Material and methods:**

50 endocervical swabs were collected from women of reproductive age group attending infertility clinic and stored at −80 ºC. DNA extraction was done with Helini bacterial mini spin kit and tested for C. trachomatis DNA by RT-PCR kit.

**Results:**

Of the 50 patients, 43 (86%) had primary infertility, and 7 (14%) had secondary infertility. Three (6%) were positive for C. trachomatis by RT-PCR. Two had primary infertility and one had secondary infertility.

**Conclusion:**

Routine screening of C. trachomatis even in high-risk populations is not available in developing countries like India. The World Health Organization recommends syndromic approach for case management. Hence, a cost-effective, highly sensitive and specific test is the pressing priority in resource poor settings.

## Introduction

According to the World Health Organization (WHO), the reason for infertility is multi-factorial, including female factors, male factors and unexplained factors in some cases. The magnitude of infertile couples all over the world was found to be 60–80 million. It has been estimated to affect 8–12% of couples globally, and this may vary in different regions of the world [1]. Female factors include ovulation disorders (25%), tubal defects (22%), endometriosis (15%), pelvic adhesion (11%) and hyperprolactinemia (7%) [2]. Genital infection due to *C. trachomatis* is one of the most prevalent sexually transmitted infections (STIs), and it has been recorded that 92 million new infections occur worldwide every year [3]. The diagnosis of *C. trachomatis* infection is difficult as approximately 70–80% of women are asymptomatic. Undiagnosed infections may present as PID, leading to ectopic pregnancy, infertility or other adverse health outcomes [4]. Further, STIs increase the risk of acquisition of HIV. With this background, this study was done to assess the prevalence of *C. trachomatis* infections among female patients with infertility using RT-PCR and to compare the findings of molecular testing with hysterosalpingography (HSG) and ultrasonography (USG).

## Material and methods

This prospective cross-sectional study was done in a tertiary care hospital. Institutional Ethics Committee Clearance was obtained from the local ethics committee-TIREC (Ref. No: 1629/MICRO/2019) and written consent had been obtained from each patient after full explanation of the purpose and nature of all procedures used. Confidentiality was maintained. Fifty endocervical swabs were collected from women of reproductive age group attending infertility clinic and stored at −80 ºC.

DNA extraction was done with the Helini bacterial mini spin kit. The elute was used for *C. trachomatis* DNA detection by RT-PCR kit containing the target sequence (OMP gene), which was a highly conserved region and previously proved to be a good genetic marker. The analytical sensitivity of the kit used was determined by analysis of dilution series of quantified *C. trachomatis* specific DNA from 0.001 copies to 10 copies/μl in triplicates. Under optimal PCR conditions, the analytical sensitivity is 0.95 copies per micro liter.

HSG was done to assess the tubal patency and USG was carried out to find out other abnormalities in all the cases.

## Inclusion criteria

– Women of reproductive age
– History of primary and secondary infertility
– Abstinence from sexual intercourse for 72 hours before sample collection
– Primary infertility refers to the couples who have not become pregnant at least once
– Secondary infertility refers to the mother who is unable to become pregnant following the birth of a child
– although not necessarily a live birth

## Exclusion criteria

– Infertility due to congenital cause
– Infertility due to male factor
– Vaginal cream application/antibiotics in the last 30 days

## Results

Of the 50 patients, 43 (86%) had primary infertility, and 7 (14%) had secondary infertility. The mean age was 28.1 years. Eighteen (36%) had PID symptoms. Screening by HSG showed tubal block in four (8%) patients. USG showed features suggestive of PID (adenexal cyst/fimbrial cyst/paratubal cyst) in 11 (22%) patients. Thirty-one (62%) patients yielded growth from endocervical swabs submitted for routine culture. Most common organisms isolated were *Candida species* (24%) followed by *Escherichia coli* (18%). Sixteen (32%) had taken antibiotics.

Three (6%) were positive for *C. trachomatis* by RT-PCR. Two had primary infertility and one had secondary infertility. All the three were in the age group 25–29 years. One had tubal block (Table 1).

**Table 1.** Distribution of *C. trachomatis* in women with infertility

**Age in years**	**RT PCR for *C. trachomatis***		**Total**	***P* value**

	**Positive**	**Negative**		
20–24	0	14	14	0.252
25–29	3	15	18	Fisher's exact test
30–34	0	13	13	
35–39	0	4	4	
>40	0	1	1	
	3	47	50	

## Discussion

STIs are responsible for 70% of PIDs [5]. PID is usually polymicrobial and recurrent episodes lead to infertility due to tubal defects. Chance of infertility increases after every episode of PID: it has been found to be 25% for the first episode, 50% following the second episode and 75% following the third episode [6].

*C. trachomatis* infection may present as primary or chronic recurrence or reinfection. Reinfection is due to repeated infection, while recurrence is due to the presence of *Chlamydia* reservoir in the lymph node or spleen. Macrophages play an important role in recurrence, as *Chlamydia* circulates within macrophages and forms a temporary shelter in the lymph node, spleen or serous cavities [7]. Atypical forms display decreased major outer membrane protein, and lipopolysaccharide antigens produce heat shock proteins, continuously leading to chronic infection [8].

Recurrence is common in young women due to the anatomic differences in the cervix; the squamocolumnar junction, which is the primary target for *C. trachomatis*, is everted and more exposed [8].

Risk factors include being unmarried, nulliparity, race, poor socio-economic status, having multiple sexual partners, lack of use of barrier contraceptive devices, concurrent gonococcal infection and use of oral contraceptive [9].

Osborne et al. recorded that 80% of patients with PID showed antibodies to *C. trachomatis* and demonstrated very high or even rising titre [10]. The other organisms causing PID are *Trichomonas vaginalis, Gardnerella vaginalis, Candida albicans, Neisseria gonorrheae, Mycoplasma* and *Mycobacteriun tuberculosis* [11].

Kulkarni et al. collected endocervical swabs from PID cases and found that 62% were aerobic culture positive. Organisms isolated were *Staphyococcus aureus* (7%), *Es.coli* (11%), *Klebsiella* (6%), *Pseudomonas* (5%), *Enterococci* (3%), *Streptococci* (3%), *Proteus* (2%), *Gonococci* (6%), *Candida* (6%) and mixed flora (1%) [11].

High prevalence of *C. trachomatis* was noted in symptomatic, sexually active women of child-bearing age and in high-risk groups. Joyee et al. noted 2.4% positivity by IgM ELISA and 1.1% in urine by PCR [12]. Treharne et al. observed 62% seroprevalence of IgG *C. trachomatis* in PID cases [13]. These antibodies persist for a longer period. Hence, acute, chronic or resolved infection cannot be differentiated. Further, cross-reacting antibodies to lipopolysaccharide antigens of other Chlamydia including *C. pneumoniae* and other gram negative LPS can produce false positive serology [14].

Most *C. trachomatis* infections are asymptomatic, and the pathogen load will be low in chronic or persistent infections, which creates challenges in the diagnosis. In addition to this, it is an intracellular organism, which makes examination more difficult [3]. Clinically it is difficult to distinguish chlamydial infection from other lower genital tract infections. Endocervical, vaginal, vulval, urethral, rectal swabs and first catch urine are common samples collected. Vulval and vaginal swabs were found to be more sensitive than urine in the detection of *C. trachomatis* infection in females. Chance of recovery is 10–20% more if both cervical and urethral specimens are collected [8].

*C. trachomatis* can be cultivated in cell lines such as McCoy, Buffalo Green Monkey (BGM), and He La-229, which is a gold standard test in the diagnosis. Nevertheless, tissue culture is a costly, laborious, time-consuming and less sensitive method to be practiced. The low sensitivity is due to the issues related to the quality, storage and processing of specimen.

Further, it is not widely available in all laboratories. Hence, an alternative, more feasible method is needed especially in resource poor settings [8].

The commonly used method is antigen detection by direct fluorescent antigen (DFA), which is 90% sensitive and 98–99% specific. High specificity depends on visualization of distinctive morphology and staining characteristics of *Chlamydia trachomatis* inclusions and elementary bodies (EB). Around 30% of clinical samples contain ≤ 10 EBs, they will be reported as negative. It is a rapid method and does not require refrigeration during transport of specimen. However, it requires a costly fluorescent microscope and expertise for interpretation [3,15].

PCR is the sensitive and specific method in the diagnosis. A low prevalence of 6% was noted in this study which reflects active infection. Similar results were noted in studies conducted in India by Sood et al. (9.28%), and Dwibedi et al. (7.04%) using PCR among symptomatic females. [16,17]. As most of the patients are treated based on syndromic management, there is low prevalence of *C. trachomatis* detected by PCR, which reflects the tip of the iceberg. Although the seroprevalence studies using IgG antibody to *C. trachomatis* revealed high prevalence, it was not included in this study.

Four patients (8%) had a tubal block detected by HSG. Of this only one was PCR positive. Quantitative RT-PCR detects the burden of organisms which may vary from 10 to over a million organisms per ml of genital tract secretions [18]. High load is associated with clinical symptoms, transmissibility, persistence of infection and complications.

### Limitations of the study

1. In the present study, the samples were tested using PCR, but the results were not compared with DFA detection which is the gold standard test for detection of *C. trachomatis*.
2. Also, the IgG antibodies to *C. trachomatis* by ELISA was not performed in the present study, which may have detected a high prevalence in the study population comparatively.

## Conclusion

This study highlights the need for the screening for infectious etiology, along with USG and HSG examination among women with infertility. Routine screening of *C. trachomatis* even in high-risk population is not available in developing countries like India. Further, there is a high burden of STIs. Hence, WHO recommends syndromic approach for case management. Initiation of antibiotic treatment is a double-edged sword. A cost effective, highly sensitive and specific test is the pressing priority in resource poor settings.
